# Correction: Mahmood et al. Aging in the Right Place for Older Adults Experiencing Housing Insecurity: An Environmental Assessment of Temporary Housing Program. *Int. J. Environ. Res. Public Health* 2022, *19*, 14857

**DOI:** 10.3390/ijerph20136260

**Published:** 2023-06-30

**Authors:** Atiya Mahmood, Rachelle Patille, Emily Lam, Diana Juanita Mora, Shreemouna Gurung, Gracen Bookmyer, Rachel Weldrick, Habib Chaudhury, Sarah L. Canham

**Affiliations:** 1 Department of Gerontology, Simon Fraser University, 515 West Hastings, Suite 2800, Vancouver, BC V6B 5K3, Canada; 2 Faculty of Health Sciences, Simon Fraser University, 8888 University Dr, Burnaby, BC V5A 1S6, Canada; 3 College of Social Work, University of Utah, 395 S 1500 E, Salt Lake City, UT 84112, USA; 4 College of Architecture and Planning, 375 1530 E, University of Utah, Salt Lake City, UT 84112, USA

**Table Correction**

In the original publication [1], there was a mistake in Table 3, only half of the table was included in the published manuscript. The correct Table 3 appears below.

**Text Correction**

There was an error in the original publication. The manuscript stated “four” analytical concepts when there are in fact five.

A correction has also been made to the Section 7, Paragraph 1 and should read:

The AIRP-ENV and AIRP-ENV SO audit tools can be used to effectively evaluate different types of temporary and permanent supportive housing across the housing continuum for OPEH. By linking the AIRP-ENV and AIRP-ENV SO tools to existing AIRP indicators, this research fills a gap in the literature on the environmental role of the AIRP on OPEH. The findings from the AIRP-ENV tool can support further identification of areas for environmental interventions at housing sites targeted for OPEH. Additionally, the tool can inform planning and design guidelines for housing that support marginalized OPEH who experience a range of individual health and functional statuses. Continued efforts in the THP to improve and adapt environmental features related to the five analytical concepts of *Functionality, Accessibility, Safety and Security, Social Activity*, and *Autonomy and Identity* are warranted. These implementations could improve the well-being and housing stability of OPEH.

The authors apologize for any inconvenience caused and state that the scientific conclusions are unaffected. The original publication has also been updated.

## Figures and Tables

**Table 3:** Overall scoring of AIRP concepts per THP building.

Overall Scoring					
Buildings	Functionality	Accessibility	Safety and Security	Social Activity	Autonomy and Identity
Hallow Place	63.6%	73.0%	41.4%	47.6%	62.5%
Arbutus Plaza	67.1%	64.2%	45.4%	49.4%	57.1%
Meadow Gardens	57.3%	59.3%	47.2%	34.4%	No Access
Sunset Place	62.2%	54.6%	60.6%	30.1%	71.4%
