# Supplementary material for: Aging in the Right Place for Older Adults Experiencing Housing Insecurity: An Environmental Assessment of Temporary Housing Program
Source: Int J Environ Res Public Health. 2022 Nov 11;19(22):14857. doi: 10.3390/ijerph192214857 (PMC9691151; doi:10.3390/ijerph192214857)
Supplement: Supplementary file 1 [file ijerph-19-14857-s001.zip › 2021.03.11_AIRP-ENV Checklist.pdf]

THE AGING IN THE RIGHT PLACE – ENVIRONMENTAL AUDIT TOOL (AIRP- ENV)

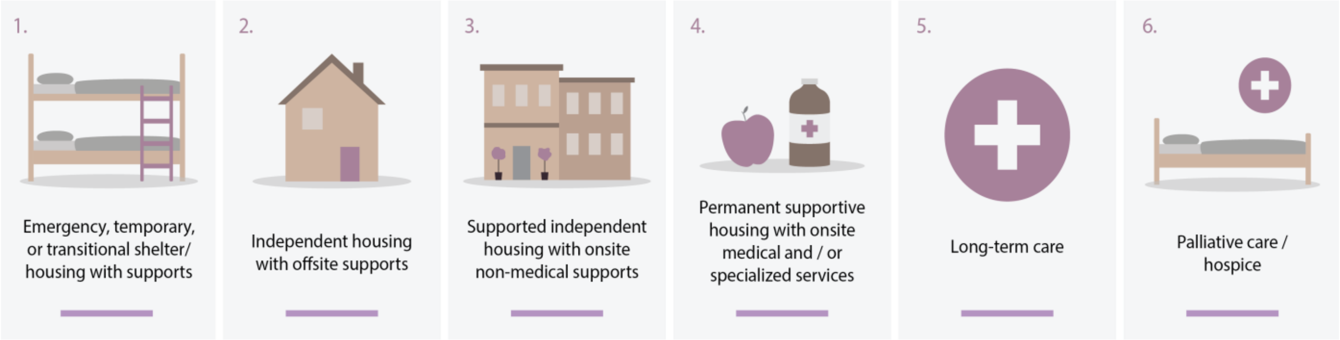

Project Title:
Aging in the Right Place: Building Capacity for Promising Practices that Support Older People Experiencing Homelessness in Montreal, Calgary, and Vancouver

Project Director:
Dr. Sarah Canham, College of Social Work, University of Utah; Department of Gerontology, Simon Fraser University (SFU), scanham@sfu.ca

Vancouver City-Lead
Dr. Atiya Mahmood, Associate Professor, Department of Gerontology, Simon Fraser University, amahmood@sfu.ca

Calgary City-Lead
Dr. Christine Walsh, Professor, Faculty of Social Work, University of Calgary, cwalsh@ucalgary.ca

Montreal City-Lead
Dr. Tamara Sussman, Associate Professor, School of Social Work, McGill University, tamara.sussman@mcgill.ca

Granting Agency:
Canadian Mortgage and Housing Corporation - Social Sciences and Humanities Research Council

---

Version 7: March 11, 2021

AGING IN THE RIGHT PLACE ENVIRONMENTAL AUDIT TOOL (AIRP- ENV)

Introduction

The Aging in the Right Place Environmental Checklist (AIRP- ENV) is an environmental audit tool that is designed for researchers to evaluate the design and accessibility of the following promising practices for individuals experiencing homelessness. This tool should be used for the following promising practice types:

1) Emergency, temporary, or transitional shelter/housing with support;
2) Independent housing with offsite supports available;
3) Supported independent housing with onsite, non-medical supports;
4) Permanent supportive housing with onsite medical and/or specialized services

This tool has been adapted from the Physical and Architectural Features Checklist (PAF), which is part of the Multiphasic Environmental Assessment Procedure (MEAP).

Date of Data Collection: ______________________________

Start Time (AM/PM): ______________ End Time (AM/PM): ______________

Break (minutes – if applicable): ______________________________

Name of Research Assistant: ______________________________

Name of Promising Practice: ______________________________

# of Building Location(s) used for Promising Practice: ______________________________

# of Buildings in Audited Housing Complex: ______________________________

Identifying Name of Audited Building: ______________________________

Address of Audited Building: ______________________________

# of Resident Rooms per Building: ______________________________

# of Type of Resident Rooms ____ STUDIO ___ ONE BEDROOM ___TWO BEDROOM ___OTHER

If Other, please specify: ______________________________

SECTION A: EXTERIOR OF THE BUILDING
Accessibility/Mobility/Safety/Security/Social Activity

Is the exterior main entrance to the building:

1. Sheltered from the sun and rain and snow?	□ Yes 1	□ No 2	
2. Is the exterior main entrance adequately lit?	□ Yes 1	□ No 2	□ N/A 97
2a. How many light fixtures are present?	#		
2b. How many light fixtures are lit/functional?	#		□ N/A 97
2c. Adequately lit at night for older adults?	□ Yes 1	□ No 2	□ N/A 97
3. Visible from seating in the lobby?	□ Yes 1	□ No 2	□ N/A 97
4. Visible from the station of an employee?	□ Yes 1	□ No 2	□ N/A 97
5. Is the entrance barrier free?	□ Yes 1	□ No 2	□ N/A 97
6. Is there outside seating in front of the building?	□ Yes 1	□ No 2	
6a. Is it visible from the entrance lobby?	□ Yes 1	□ No 2	□ N/A 97
6b. Is it visible from a ground floor social space?	□ Yes 1	□ No 2	□ N/A 97
6c. Is it visible from the station of an employee?	□ Yes 1	□ No 2	□ N/A 97
6d. Is it protected from the weather?	□ Yes 1	□ No 2	□ N/A 97
6e. Is it provided with a view of pedestrians, etc.?	□ Yes 1	□ No 2	□ N/A 97

7. Is there a communal patio or courtyard or open space for the use of the residents?	□ Yes 1	□ No 2	
7a. Is there seating for residents (uncovered)?	□ Yes 1	□ No 2	□ N/A 97
7a.i. Small group seating area (e.g. 2-3 people)?	□ Yes 1	□ No 2	□ N/A 97
7a.ii. Large group seating area (6 or more people)?	□ Yes 1	□ No 2	□ N/A 97
7b. Are tables available?	□ Yes 1	□ No 2	□ N/A 97
7c. Are umbrella tables available?	□ Yes 1	□ No 2	□ N/A 97
7d. Is the outdoor furniture in good condition?	□ Yes 1	□ No 2	□ N/A 97
7d.i. Is the outdoor furniture barrier-free?	□ Yes 1	□ No 2	□ N/A 97
7e. Is there a covered/rainproof area?	□ Yes 1	□ No 2	□ N/A 97
7e.i. Is there a seating area?	□ Yes 1	□ No 2	□ N/A 97
7f. Is there an area with protection from the sun?	□ Yes 1	□ No 2	□ N/A 97
7g. Is there a barbecue?	□ Yes 1	□ No 2	□ N/A 97
7h. Is there a greenhouse?	□ Yes 1	□ No 2	□ N/A 97
7i. Is there an area for residents to do gardening?	□ Yes 1	□ No 2	□ N/A 97
7j. Are there raised garden beds?	□ Yes 1	□ No 2	□ N/A 97
7k. Is there a lawn/green space?	□ Yes 1	□ No 2	□ N/A 97
7l. Is there a landscaped area in which residents can sit or stroll?	□ Yes 1	□ No 2	□ N/A 97

7l.i. Is the landscaped area well-maintained?<br>7l.ii. Are there dedicated walking paths?<br>7l.iii. Are the paths looped?<br>7m. Is it accessible to people using wheelchairs or walkers?<br>7n. Is this communal patio or courtyard adequately lit?	□ Yes 1<br>□ Yes 1<br>□ Yes 1<br>□ Yes 1<br>□ Yes 1	□ No 2<br>□ No 2<br>□ No 2<br>□ No 2<br>□ No 2	□ N/A 97<br>□ N/A 97<br>□ N/A 97<br>□ N/A 97<br>□ N/A 97
7n.i. How many light fixtures are present?	#		
7n.ii. How many light fixtures are lit/functional?	#		□ N/A 97
7n.iii. Adequately lit at night for older adults?	□ Yes 1	□ No 2	□ N/A 97
8. Are there other outdoor recreational amenities available (e.g. shuffleboard, golf putting, etc.)?	□ Yes 1	□ No 2	□ N/A 97
8a. If YES, please describe:			
9. Are there places for residents to preview the outdoor space? (e.g. places from where one can view to observe before participating)	□ Yes 1	□ No 2	□ N/A 97
10. Are there windows overlooking the outdoor space?	□ Yes 1	□ No 2	□ N/A 97
11. Is there fencing around the outdoor space?	□ Yes 1	□ No 2	□ N/A 97
12. Is the outdoor space secure?<br>(e.g., perimeter controlled, so outside people cannot easily enter)	□ Yes 1	□ No 2	□ N/A 97
13. Is there a staff indoor surveillance option of the outdoor space?	□ Yes 1	□ No 2	□ N/A 97
14. Is the access to the outdoor space locked?	□ Yes 1	□ No 2	□ N/A 97
15. Is the access to outdoor space available to residents during regular hours? (e.g. 9am – 5pm or 8am – 8pm)	□ Yes 1	□ No 2	□ N/A 97

With regard to the landscaping around the site:

16. Does it contain a variety of natural plantings?<br>16a. Flowering plants present?<br>16b. Fruit bearing plants present?	□ Yes 1<br>□ Yes 1<br>□ Yes 1	□ No 2<br>□ No 2<br>□ No 2	 □ N/A 97<br>□ N/A 97
17. Are there paths/walkways?	□ Yes 1	□ No 2	
17a. Are the paths/walkways barrier-free?	□ Yes 1	□ No 2	□ N/A 97
17b. Are there ramps leading to and from paths/walkways?	□ Yes 1	□ No 2	□ N/A 97
18. Are the mature trees and shrubs (i.e. established/large)?	□ Yes 1	□ No 2	
19. Are there trees that provide a shaded area?	□ Yes 1	□ No 2	
20. Are the gardens/grounds well-maintained?	□ Yes 1	□ No 2	
21. Are the gardens/grounds adequately lit?	□ Yes 1	□ No 2	
21a. How many light fixtures are present?	#		
21b. How many light fixtures are lit/functional?	#		□ N/A 97
21c. Adequately lit at night for older adults?	□ Yes 1	□ No 2	□ N/A 97
22. Is designated parking available?<br>*(Note: If NO, skip to Q23)*	□ Yes 1	□ No 2	□ N/A 97

22a. For residents?<br>22b. For handicapped?<br>22c. For staff?<br>22d. For visitors?	□ Indoor 1<br>□ Indoor 1<br>□ Indoor 1<br>□ Indoor 1	□ Outdoor 1a<br>□ Outdoor 1a<br>□ Outdoor 1a<br>□ Outdoor 1a	□ No 2<br>□ No 2<br>□ No 2<br>□ No 2	□ N/A 97<br>□ N/A 97<br>□ N/A 97<br>□ N/A 97

22e. How many parking spaces are there?<br>*(Note: Clarify types of parking available in Q27)*	#<br>Indoor 1	#<br>Outdoor 1a	□ N/A 97
23. Is there secure bicycle storage?<br>23a. If yes, indoor or outdoor?	□ Yes 1<br>□ Indoor 1	□ No 2<br>□ Outdoor 1a	□ N/A 97<br>□ N/A 97
24. Is there secure scooter storage?<br>24a. If yes, indoor or outdoor?	□ Yes 1<br>□ Indoor 1	□ No 2<br>□ Outdoor 1a	□ N/A 97<br>□ N/A 97

25. Is the parking adequately lit?	□ Yes 1	□ No 2	□ N/A 97
25a. How many light fixtures are present?	#		
25b. How many light fixtures are lit/functional?	#		□ N/A 97
25c. Adequately lit at night for older adults?	□ Yes 1	□ No 2	□ N/A 97
26. Is there signage showing actions taken for COVID-19 safety protocols?	□ Yes 1	□ No 2	□ N/A 97
26a. Do the protocols impact access to outdoor spaces?	□ Yes 1	□ No 2	□ N/A 97
26b. Do the protocols impact direction of walking on pathways?	□ Yes 1	□ No 2	□ N/A 97
26c. Do the protocols impact parking availability?	□ Yes 1	□ No 2	□ N/A 97
26d. Are the protocols posted in multiple languages?	□ Yes 1	□ No 2	□ N/A 97
26d.i. If yes, please note the languages below:			□ N/A 97

27. NOTES/COMMENTS:

SECTION B: INTERIOR OF THE BUILDING – COMMUNAL/SOCIAL/RECREATIONAL AND CIRCULATION SPACES

Accessibility/Safety/Security/Social Activity/Belonging

PART 1: LOBBY AND ENTRANCE AREA (CIRCULATION AND GATHERING)

Exterior entrance to the building:

28. Can one enter the building from the street without having to use any stairs?	□ Yes 1	□ No 2	
29. Is the entry from outside limited to one unlocked door?	□ Yes 1	□ No 2	
30. Is there a bell or call system at the front entrance?	□ Yes 1	□ No 2	
30a. Does the bell or call system have a light signal?	□ Yes 1	□ No 2	□ N/A 97
31. Are written instructions posted outside that explain how to get in if the front door is locked?	□ Yes 1	□ No 2	
32. Is the front door open by a buzzer system in each suite?	□ Yes 1	□ No 2	
33. Does the front door open automatically?	□ Yes 1	□ No 2	
34. Does the front door have a lever or round handle?	□ (Lever 1)	□ (Round 1a)	□ (Other 1b)
34a. If Other, please specify:			
35. Does the front door swing closed by itself?	□ Yes 1	□ No 2	
36. Is the front door wide enough for a wheelchair?	□ Yes 1	□ No 2	

Lobby or entrance area within the building:

37. Is access to the building monitored (unrelated to COVID-19)?	□ Yes 1	□ No 2	□ N/A 97
38. Is there a reception area or reception desk?	□ Yes 1	□ No 2	□ N/A 97
39. Is there a place for visitors to sign in?	□ Yes 1	□ No 2	□ N/A 97
40. Is there an area for posters and notices?	□ Yes 1	□ No 2	□ N/A 97
41. Is there an adjacent room for a visiting doctor?	□ Yes 1	□ No 2	□ N/A 97
42. Is there a washroom nearby?	□ Yes 1	□ No 2	

43. Is there a pick-up mail area?	□ Yes 1	□ No 2	
44. Is there visible signage for after-hours emergency contact numbers?	□ Yes 1	□ No 2	□ N/A 97
45. Does this space have natural light?	□ Yes 1	□ No 2	
45a. If so, how many windows?	#		
45b. Do the windows open and close to allow ventilation?	□ Yes 1	□ No 2	□ N/A 97
46. Does this space have artificial light?	□ Yes 1	□ No 2	
46a. How many light fixtures are present?	#		
46b. How many light fixtures are lit/functional?	#		□ N/A 97

47. Are baseboard heaters present?<br>47a. Fireplaces?<br>*(Note: If 'NO' for baseboard or fireplace heating, note the type of heating system used in Q54)*	□ Yes 1<br>□ Yes 1	□ No 2<br>□ No 2	□ N/A 97<br>□ N/A 97
48. Does the building have a lobby?<br>48a. Is there seating in the lobby?<br>48b. Is the seating barrier-free?<br>48c. Is the seating facing or angled toward each other?<br>48d. Is there a large face clock in the lobby area?<br>48e. Is there access to power outlets?<br>48f. Is there signage for available Wi-Fi?	□ Yes 1<br>□ Yes 1<br>□ Yes 1<br>□ Yes 1<br>□ Yes 1<br>□ Yes 1<br>□ Yes 1	□ No 2<br>□ No 2<br>□ No 2<br>□ No 2<br>□ No 2<br>□ No 2<br>□ No 2	 □ N/A 97<br>□ N/A 97<br>□ N/A 97<br>□ N/A 97<br>□ N/A 97<br>□ N/A 97
48g. Approximately what size is the lobby?	sq. m. or sq. ft.		
49. Is there a lounge near the entrance (other than the lobby)?<br>49a. Is the lounge furnished?<br>49b. Do the furnishings seem comfortable for residents?<br>49c. Are the furnishings barrier-free?<br>49d. Are the furnishings/finishes homelike?<br>49e. Does the décor reflect a specific theme?<br>49f. Is the lobby or entrance area visible from the lounge or other ground floor social space?	□ Yes 1<br>□ Yes 1<br>□ Yes 1<br>□ Yes 1<br>□ Yes 1<br>□ Yes 1<br>□ Yes 1	□ No 2<br>□ No 2<br>□ No 2<br>□ No 2<br>□ No 2<br>□ No 2<br>□ No 2	□ N/A 97<br>□ N/A 97<br>□ N/A 97<br>□ N/A 97<br>□ N/A 97<br>□ N/A 97<br>□ N/A 97
50. Does the lounge have natural light?	□ Yes 1	□ No 2	□ N/A 97
50a. If so, how many windows?	#		
50b. Do the windows open and close to allow ventilation?	□ Yes 1	□ No 2	□ N/A 97
51. Does this space have artificial light?	□ Yes 1	□ No 2	□ N/A 97

51a. How many light fixtures are present?	#		
51b. How many light fixtures are lit/functional?	#		□ N/A 97
52. Is there artwork displayed in the lobby?<br>52a. Does the artwork follow a theme/cultural acknowledgement?	□ Yes 1<br>□ Yes 1	□ No 2<br>□ No 2	 □ N/A 97
52b. If yes, please explain:			
53. Is there signage showing actions taken for COVID-19 safety protocols?<br>53a. Do the protocols impact use of furnished seating in the lobby?<br>53b. Do the protocols impact capacity in the lobby?<br>53c. Are the protocols posted in multiple languages?<br>53c.i. If yes, please note the languages below:	□ Yes 1<br>□ Yes 1<br>□ Yes 1<br>□ Yes 1	□ No 2<br>□ No 2<br>□ No 2<br>□ No 2	□ N/A 97<br>□ N/A 97<br>□ N/A 97<br>□ N/A 97<br>□ N/A 97

54. NOTES/COMMENTS:

PART 2: HALLS AND STAIRWAY AREA (CIRCULATION SPACE)
Accessibility/Safety

Are the hallways:

55. How wide are the hallways in meters or feet?	m. or ft.

56. Crowded or obstructed (e.g. wheelchairs, cleaning equipment)?	□ Yes 1	□ No 2	
57. Equipped with handrails?<br>57a. Are there half-dome/ convex safety mirrors?	□ Yes 1<br>□ Yes 1	□ No 2<br>□ No 2	
58. Decorated, e.g. with pictures or plants?	□ Yes 1	□ No 2	
59. Equipped with smoke detection devices?<br>59a. Fire extinguishers?<br>59b. Carbon Monoxide detectors?<br>59c. Sprinkler system?	□ Yes 1<br>□ Yes 1<br>□ Yes 1<br>□ Yes 1	□ No 2<br>□ No 2<br>□ No 2<br>□ No 2	
60. Adequately lighted?	□ Yes 1	□ No 2	
60a. How many light fixtures are present?	#		
60b. How many light fixtures are lit/functional?	#		□ N/A 97
61. Does this space have natural light?	□ Yes 1	□ No 2	
61a. If so, how many windows?	#		
61b. Do the windows open and close to allow ventilation?	□ Yes 1	□ No 2	□ N/A 97
62. Are there drinking fountains?<br>62a. Are they accessible to wheelchair residents?	□ Yes 1<br>□ Yes 1	□ No 2<br>□ No 2	 □ N/A 97
62b. How many are there per floor?	#		□ N/A 97
63. Are there public telephones?<br>63a. Is there one accessible to wheelchair residents?<br>63b. Does one have volume control for the hard of hearing?<br>63c. Is there a writing surface by the telephone?<br>63d. Is there a public phone on every floor?	□ Yes 1<br>□ Yes 1<br>□ Yes 1<br>□ Yes 1<br>□ Yes 1	□ No 2<br>□ No 2<br>□ No 2<br>□ No 2<br>□ No 2	□ N/A 97<br>□ N/A 97<br>□ N/A 97<br>□ N/A 97<br>□ N/A 97
63e. How many public phones are there per floor?	#		□ N/A 97

64. Must any stairs be climbed in order to have access to any areas for common resident use?	□ Yes 1	□ No 2	
64a. If YES, where are they located?			□ N/A 97
65. Are there elevators to provide access to other floors?<br>65a. Wide enough to accommodate wheelchair or scooter?	□ Yes 1<br>□ Yes 1	□ No 2<br>□ No 2	□ N/A 97<br>□ N/A 97

<u>Including fire exit stairs:</u>

66. Is there a fire alarm with sound?<br>66a. Is there fire alarm with visual signaling (e.g. light)?	□ Yes 1<br>□ Yes 1	□ No 2<br>□ No 2	 □ N/A 97
67. Is there a Security System in the fire exit stairwell?	□ Yes 1	□ No 2	
67a. Does the Security System have cameras?	□ Yes 1	□ No 2	□ N/A 97
67b. Does the alarm have an auditory signal?	□ Yes 1	□ No 2	□ N/A 97
68. Do the stairs look safe?	□ Yes 1	□ No 2	
69. Are the stairs well lighted?	□ Yes 1	□ No 2	
69a. How many light fixtures are present?	#		
69b. How many light fixtures are lit/functional?	#		□ N/A 97
70. Does this space have natural light?	□ Yes 1	□ No 2	
70a. If so, how many windows?	#		
70b. Do the windows open and close to allow ventilation?	□ Yes 1	□ No 2	□ N/A 97
71. Are there non-skid surfaces on stairs and ramps?	□ Yes 1	□ No 2	

72. Are there appropriate handrails?	□ Yes 1	□ No 2	
73. Do the handrails contrast with the wall colour?	□ Yes 1	□ No 2	
74. Are there tactile warnings at changes in level?	□ Yes 1	□ No 2	

<u>For the ease of orientation within the building:</u>

75. Is the floor/corridor adequately colour-coded?	□ Yes 1	□ No 2	
76. Is each floor adequately numbered?	□ Yes 1	□ No 2	
77. Are residents' names on or next to their doors?	□ Yes 1	□ No 2	
78. Does the building have easily readable signage?	□ Yes 1	□ No 2	
79. Is the building small and easy to navigate?	□ Yes 1	□ No 2	
80. Is wayfinding easy?	□ Yes 1	□ No 2	
80a. Has wayfinding been impacted by COVID-19 protocols?	□ Yes 1	□ No 2	□ N/A 97
80b. If so, please describe:			
81. Is there a bulletin board in a public location?	□ Yes 1	□ No 2	
82. Is there an audible sound system or public address system?	□ Yes 1	□ No 2	

83. NOTES/COMMENTS:

PART 3: COMMUNAL AND RECREATIONAL AREAS (SOCIAL ACTIVITY & GATHERING SPACES)

Social Activity/Comfort/Belonging/Health

What kinds of communal rooms and recreational or special activity areas are there:
(Check and complete only for the existing communal rooms)

84. What types of recreational or special activity materials are available? (check all that apply)			
84a. Pool or billiard table?	□ Yes 1	□ No 2	□ N/A 97
84b. Ping-pong table?	□ Yes 1	□ No 2	□ N/A 97
84c. Piano or organ?	□ Yes 1	□ No 2	□ N/A 97
84d. One or more radios?	□ Yes 1	□ No 2	□ N/A 97
84e. One or more sewing machines?	□ Yes 1	□ No 2	□ N/A 97
84f. Televisions?	□ Yes 1	□ No 2	□ N/A 97
84g. Computers?	□ Yes 1	□ No 2	□ N/A 97
84h. Board Games/Puzzles?	□ Yes 1	□ No 2	□ N/A 97
84i. Other (please specify in blank spaces below):			□ N/A 97

85. Library/Reading Room?	□ Yes 1	□ No 2	
85a. Secondary Function (if applicable)?			□ N/A 97
85b. Size?			sq. m. or sq. ft.
85c. Capacity (if visually available)?			□ N/A 97
85d. Wheelchair Accessible?	□ Yes 1	□ No 2	
85e. Additional Comments (e.g. equipment, etc.)?			

86. Music Room?	□ Yes 1	□ No 2	
86a. Secondary Function (if applicable)?			□ N/A 97
86b. Size?	sq. m. or sq. ft.		
86c. Capacity (if visually available)?			□ N/A 97
86d. Wheelchair Accessible?	□ Yes 1	□ No 2	
86e. Additional Comments (e.g. equipment, etc.)?			

87. Games Room?	□ Yes 1	□ No 2	
87a. Secondary Function (if applicable)?			□ N/A 97
87b. Size?	sq. m. or sq. ft.		
87c. Capacity (if visually available)?			□ N/A 97
87d. Wheelchair Accessible?	□ Yes 1	□ No 2	
87e. Additional Comments (e.g. equipment, etc.)?			

88. Exercise Room?	□ Yes 1	□ No 2	
88a. Secondary Function (if applicable)?			□ N/A 97
88b. Size?	sq. m. or sq. ft.		
88c. Capacity (if visually available)?			□ N/A 97
88d. Wheelchair Accessible?	□ Yes 1	□ No 2	

88e. Additional Comments (e.g. equipment, etc.)?	

89. Arts and Crafts Room?	□ Yes 1	□ No 2	
89a. Secondary Function (if applicable)?			□ N/A 97
89b. Size?	sq. m. or sq. ft.		
89c. Capacity (if visually available)?			□ N/A 97
89d. Wheelchair Accessible?	□ Yes 1	□ No 2	
89e. Additional Comments (e.g. equipment, etc.)?			

90. Prayer/Meditation Room?	□ Yes 1	□ No 2	
90a. Secondary Function (if applicable)?			□ N/A 97
90b. Size?	sq. m. or sq. ft.		
90c. Capacity (if visually available)?			□ N/A 97
90d. Wheelchair Accessible?	□ Yes 1	□ No 2	
90e. Additional Comments (e.g. equipment, etc.)?			

91. Community Room?	□ Yes 1	□ No 2	
91a. Secondary Function (if applicable)?			□ N/A 97
91b. Size?	sq. m. or sq. ft.		
91c. Capacity (if visually available)?			□ N/A 97
91d. Wheelchair Accessible?	□ Yes 1	□ No 2	
91e. Additional Comments (e.g. equipment, etc.)?			

92. Designated Smoking Space?	□ Yes 1	□ No 2	
92a. Secondary Function (if applicable)?			□ N/A 97
92b. Outdoor or Indoor?	□ Indoor 1	□ Outdoor 1a	□ N/A 97
92c. Size?	sq. m. or sq. ft.		
92d. Capacity (if visually available)?			□ N/A 97
92e. Wheelchair Accessible?	□ Yes 1	□ No 2	
92f. Additional Comments (e.g. special ventilation, locked cabinets, equipment, etc.)?			

93. Main lounge?	□ Yes 1	□ No 2	
93a. Secondary Function (if applicable)?			□ N/A 97
93b. Size?	sq. m. or sq. ft.		
93c. Capacity (if visually available)?			□ N/A 97
93d. Wheelchair Accessible?	□ Yes 1	□ No 2	

93e. Additional Comments (e.g. equipment, etc.)?	

94. TV Lounge?	□ Yes 1	□ No 2	
94a. Secondary Function (if applicable)?			□ N/A 97
94b. Size?	sq. m. or sq. ft.		
94c. Capacity (if visually available)?			□ N/A 97
94d. Wheelchair Accessible?	□ Yes 1	□ No 2	
94e. Additional Comments (e.g. equipment, etc.)?			

95. Altogether, how many lounges are there (count)?	#	□ N/A 97
96. Where are the lounges located?		□ N/A 97

97. If the building is more than one story, are there lounges on each floor?	□ Yes 1	□ No 2	□ N/A 97
98. Are the lounges accessible at all times?	□ Yes 1	□ No 2	□ N/A 97
98a. When is it/are they accessible?			□ N/A 97
98b. Note if any of the lounges are locked and timeslots when they cannot be used:			

Within the lounges:

99. Are there seating areas?	□ Yes 1	□ No 2	□ N/A 97
99a. Is the seating area arranged in a way to allow for social interaction (e.g. facing each other or at an angle?)	□ Yes 1	□ No 2	
100. Are there folding tables?	□ Yes 1	□ No 2	□ N/A 97
101. Are there writing desks or tables?	□ Yes 1	□ No 2	□ N/A 97
102. Is reading material available on tables or shelves?	□ Yes 1	□ No 2	□ N/A 97
103. Are there table lamps?	□ Yes 1	□ No 2	□ N/A 97
104. Is furniture spaced wide enough for wheelchairs?	□ Yes 1	□ No 2	□ N/A 97
105. Is there a quiet lounge with no television?	□ Yes 1	□ No 2	□ N/A 97
106. Is the décor and furnishing home-like?	□ Yes 1	□ No 2	□ N/A 97
107. Is the lighting adequate?	□ Yes 1	□ No 2	
107a. How many light fixtures are present?	#		
107b. How many light fixtures are lit/functional?	#		□ N/A 97
108. Does this space have natural light?	□ Yes 1	□ No 2	□ N/A 97
108a. If so, how many windows?	#		
108b. Do the windows open and close to allow ventilation?	□ Yes 1	□ No 2	□ N/A 97
109. Are baseboard heaters present?<br>109a. Fireplaces?<br><br>*(Note: If 'NO' for baseboard or fireplace heating, note the type of heating system used in Q117)*	□ Yes 1<br>□ Yes 1	□ No 2<br>□ No 2	□ N/A 97<br>□ N/A 97
110. Is there cupboard space?	□ Yes 1	□ No 2	□ N/A 97
111. Is there chair storage?	□ Yes 1	□ No 2	□ N/A 97
112. Is there signage for available Wi-Fi?	□ Yes 1	□ No 2	□ N/A 97
113. Is there access to power outlets?	□ Yes 1	□ No 2	□ N/A 97
114. Are there washrooms nearby?	□ Yes 1	□ No 2	□ N/A 97

115. Are there any special or innovative features within any of the recreational areas?	□ Yes 1	□ No 2	
115a. If YES, please describe:			
116. Is there signage showing actions taken for COVID-19 safety protocols?	□ Yes 1	□ No 2	□ N/A 97
116a. Do the safety protocols impact access to social and communal areas?	□ Yes 1	□ No 2	□ N/A 97
116b. Do the safety protocols impact capacity in lounge spaces?	□ Yes 1	□ No 2	□ N/A 97
116c. Are the safety protocols posted in multiple languages?	□ Yes 1	□ No 2	□ N/A 97
116c.i. If yes, please note the languages below:			□ N/A 97

117. NOTES/COMMENTS:

PART 4: DINING AREAS (SHARED MEAL PREPARATION & DINING SPACE)

118. Communal Kitchen?	□ Yes 1	□ No 2	
118a. Secondary Function (if applicable)?			□ N/A 97
118b. Size?	sq. m. or sq. ft.		
118c. Capacity (if visually available)?			□ N/A 97
118d. Wheelchair Accessible?	□ Yes 1	□ No 2	
118e. Additional Comments (e.g. equipment, etc.)?			

119. Dining Room? *(Note: If NO, skip to Q134)*	□ Yes 1	□ No 2	
119a. Secondary Function (if applicable)?			□ N/A 97
119b. Size?	sq. m. or sq. ft.		
119c. Capacity (if visually available)?			□ N/A 97
119d. Wheelchair Accessible?	□ Yes 1	□ No 2	
119e. Additional Comments (e.g. equipment, etc.)?			

120. How many dining areas in the building?	#		□ N/A 97
121. What is the size of the smallest dining room?	sq m.		
122. What is the size of the largest dining room?	sq m.		
123. How large are these areas all together?	sq m.		
124. Would you describe the dining room furnishing/finishes as home-like?	□ Yes 1	□ No 2	□ N/A 97
125. Are there tables that seat two?	□ Yes 1	□ No 2	

126. Are there tables that seat four?	□ Yes 1	□ No 2	
127. Are there tables that seat six?	□ Yes 1	□ No 2	
128. Are there large tables that seat more than six?	□ Yes 1	□ No 2	
129. Is aisle space between tables at least 152 cm?	□ Yes 1	□ No 2	□ N/A 97
130. Is the lighting adequate?	□ Yes 1	□ No 2	
130a. How many light fixtures are present?	#		
130b. How many light fixtures are lit/functional?	#		□ N/A 97
131. Does this space have natural light?	□ Yes 1	□ No 2	
131a. If so, how many windows?	#		
131b. Do the windows open and close to allow ventilation?	□ Yes 1	□ No 2	□ N/A 97
132. Are baseboard heaters present?<br>132a. Fireplaces?<br>*(Note: If 'NO' for baseboard or fireplace heating, note the type of heating system used in Q134)*	□ Yes 1<br>□ Yes 1	□ No 2<br>□ No 2	□ N/A 97<br>□ N/A 97
133. Is there signage showing actions taken for COVID-19 safety protocols?<br>133a. Do the protocols impact number of people to a single table?<br>133b. Do the protocols impact spacing between tables?<br>133c. Do the protocols impact capacity in dining rooms?<br>133d. Are the protocols posted in multiple languages?<br>133d.i. If yes, please note the languages below:	□ Yes 1<br><br>□ Yes 1<br>□ Yes 1<br>□ Yes 1<br>□ Yes 1	□ No 2<br><br>□ No 2<br>□ No 2<br>□ No 2<br>□ No 2	□ N/A 97<br><br>□ N/A 97<br>□ N/A 97<br>□ N/A 97<br>□ N/A 97<br>□ N/A 97

134. NOTES/COMMENTS:

PART 5: SPECIAL ACTIVITIES (HEALTHCARE & SUPPORT SERVICES)

135. Doctors Room?	□ Yes 1	□ No 2	
135a. Secondary Function (if applicable)?			□ N/A 97
135b. Size?	sq. m. or sq. ft.		
135c. Capacity (if visually available)?			□ N/A 97
135d. Wheelchair Accessible?	□ Yes 1	□ No 2	
135e. Additional Comments (e.g. equipment, etc.)?			

136. Harm Reduction Space?	□ Yes 1	□ No 2	
136a. Secondary Function (if applicable)?			□ N/A 97
136b. Size?	sq. m. or sq. ft.		
136c. Capacity (if visually available)?			□ N/A 97
136d. Wheelchair Accessible?	□ Yes 1	□ No 2	
136e. Additional Comments (e.g. special ventilation, locked cabinets, equipment, etc.)?			

137. Common Laundry Room? *(Note: If NO, skip to Q140)*	□ Yes 1	□ No 2	
137a. Secondary Function (if applicable)?			□ N/A 97
137b. Size?	sq. m. or sq. ft.		
137c. Capacity (if visually available)?			□ N/A 97
137d. Wheelchair Accessible?	□ Yes 1	□ No 2	
137e. How many machines are available?	# Washers	# Dryers	
137f. Additional Comments (e.g. equipment, etc.)?			

138. Where in the housing facility are the laundry facilities located?			
138a. One laundry room on main floor?	□ Yes 1	□ No 2	□ N/A 97
138b. On each floor?	□ Yes 1	□ No 2	□ N/A 97
138c. On alternate floors?	□ Yes 1	□ No 2	□ N/A 97
138d. Other? (please specify)			
139. Are the communal machines coin-operated?<br>139a. Are they card operated?<br>139b. Is there a change machine accessible in a communal space?	□ Yes 1<br>□ Yes 1<br>□ Yes 1	□ No 2<br>□ No 2<br>□ No 2	□ N/A 97<br>□ N/A 97<br>□ N/A 97

140. Computer Room?	□ Yes 1	□ No 2	
140a. Secondary Function (if applicable)?			□ N/A 97
140b. Size?			sq. m. or sq. ft.
140c. Capacity (if visually available)?			□ N/A 97
140d. Wheelchair Accessible?	□ Yes 1	□ No 2	
140e. Additional Comments (e.g. signage for Wi-Fi, equipment, etc.)?			

141. Is there signage showing actions taken for COVID-19 safety protocols?<br>141a. Do the protocols impact access to social and communal areas?<br>141b. Do the protocols impact capacity in lounge spaces?<br>141c. Are the protocols posted in multiple languages?<br>141c.i. If yes, please note the languages below:	□ Yes 1<br>□ Yes 1<br>□ Yes 1<br>□ Yes 1	□ No 2<br>□ No 2<br>□ No 2<br>□ No 2	□ N/A 97<br>□ N/A 97<br>□ N/A 97<br>□ N/A 97<br>□ N/A 97

142. NOTES/COMMENTS:

PART 6: STAFF AND OFFICE AREAS

Safety/Social Relationships

143. Is there office space for:<br>143a. The administrative staff?<br>143b. The secretarial and clerical staff?<br>143c. Social services and counseling staff?	 □ Yes 1<br>□ Yes 1<br>□ Yes 1	 □ No 2<br>□ No 2<br>□ No 2	 □ N/A 97<br>□ N/A 97<br>□ N/A 97
143d. Other staff? (please note in spaces below)<br>(e.g. volunteers, part-time staff, janitorial staff, etc.)	□ Yes 1	□ No 2	□ N/A 97
	□ Yes 1	□ No 2	□ N/A 97
	□ Yes 1	□ No 2	□ N/A 97
	□ Yes 1	□ No 2	□ N/A 97
144. Are the offices free of distractions from adjacent activities?<br>144a. Do the offices have doors that close (for privacy)?<br>144b. Are the office doors lockable?<br>144c. Are the offices/ nearby hallways noisy?<br>144d. Are the office floors carpeted?	□ Yes 1<br>□ Yes 1<br>□ Yes 1<br>□ Yes 1<br>□ Yes 1	□ No 2<br>□ No 2<br>□ No 2<br>□ No 2<br>□ No 2	□ N/A 97<br>□ N/A 97<br>□ N/A 97<br>□ N/A 97<br>□ N/A 97
145. Are there additional rooms for:<br>145a. Handling mail, copying, or printing?<br>145b. A conference room?	 □ Yes 1<br>□ Yes 1	 □ No 2<br>□ No 2	 □ N/A 97<br>□ N/A 97
146. Is there a staff lounge?<br>146a. Is so, does it have tables?<br>146b. Does it have comfortable chairs?	□ Yes 1<br>□ Yes 1<br>□ Yes 1	□ No 2<br>□ No 2<br>□ No 2	□ N/A 97<br>□ N/A 97<br>□ N/A 97
146c. What size is it?	sq. m. or sq. ft.		□ N/A 97
147. Do these spaces function well? (Ask staff)	□ Yes 1	□ No 2	□ N/A 97
147a. If NO, why not?			
148. Is there signage showing actions taken for COVID-19 safety protocols?<br>148a. Do the protocols impact number of staff to an office/room?<br>148b. Are the protocols posted in multiple languages?	□ Yes 1<br>□ Yes 1<br>□ Yes 1	□ No 2<br>□ No 2<br>□ No 2	□ N/A 97<br>□ N/A 97<br>□ N/A 97

148b.i. If yes, please note the languages below:			□ N/A 97

149. NOTES/COMMENTS:

PART 7: SHARED BATHROOM AND TOILET AREAS

Safety/Accessibility

150. Are there any shared bathroom areas?	☐ Yes 1	☐ No 2	☐ N/A 97
150a. If YES, where in the housing complex are they located?			

151. Are they wheelchair accessible?	☐ All/Most 1	☐ Some 2	☐ Few 3	☐ No/None 4	☐ N/A 97
152. Are there raised thresholds at the entrance?	☐ All/Most 1	☐ Some 2	☐ Few 3	☐ No/None 4	☐ N/A 97
153. Do the bathroom doors open out?	☐ All/Most 1	☐ Some 2	☐ Few 3	☐ No/None 4	☐ N/A 97
154. Are there handrails or safety bars?	☐ All/Most 1	☐ Some 2	☐ Few 3	☐ No/None 4	☐ N/A 97
155. Are there lift bars next to the toilet?	☐ All/Most 1	☐ Some 2	☐ Few 3	☐ No/None 4	☐ N/A 97
156. Are the towel racks and dispensers higher than 102 cm from the floor?	☐ All/Most 1	☐ Some 2	☐ Few 3	☐ No/None 4	☐ N/A 97
157. Are there mirrors in the bathroom?	☐ All/Most 1	☐ Some 2	☐ Few 3	☐ No/None 4	☐ N/A 97
158. Are they wheelchair accessible?	☐ All/Most 1	☐ Some 2	☐ Few 3	☐ No/None 4	☐ N/A 97
159. Do areas subject to wetness have non-slip surfaces?	☐ All/Most 1	☐ Some 2	☐ Few 3	☐ No/None 4	☐ N/A 97
160. Are there call buttons?	☐ All/Most 1	☐ Some 2	☐ Few 3	☐ No/None 4	☐ N/A 97
161. Is there adequate lighting?	☐ All/Most 1	☐ Some 2	☐ Few 3	☐ No/None 4	☐ N/A 97

161a. How many light fixtures are present?	#		
161b. How many light fixtures are lit/functional?	#		☐ N/A 97
162. Does the communal bathrooms have natural light?	☐ Yes 1	☐ No 2	☐ N/A 97
162a. If so, how many windows per bathroom?	#		
162b. Do the windows open and close to allow ventilation?	☐ Yes 1	☐ No 2	☐ N/A 97

163. What kind of lighting is there in the shared bathroom areas?	☐ Incandescent 1	☐ Fluorescent 2	☐ Daylight 3	☐ N/A 97

164. What is the size of the smallest bathroom?	sq. m. or sq. ft.		☐ N/A 97
165. What is the size of the largest bathroom?	sq. m. or sq. ft.		☐ N/A 97
166. Are the bathrooms impacted by COVID-19 safety protocols?	☐ Yes 1	☐ No 2	☐ N/A 97
166a. Are the protocols posted in multiple languages?	☐ Yes 1	☐ No 2	☐ N/A 97
166a.i. If yes, please note the languages below:			☐ N/A 97

167. NOTES/COMMENTS:

PART 8: OTHER COMMON AREAS

If there are no other spaces, SKIP to question #171.

168. Other (specify primary function):			☐ N/A 97
168a. Secondary Function (if applicable)?			☐ N/A 97
168b. Size?	sq. m. or sq. ft.		
168c. Capacity (if visually available)?			☐ N/A 97
168d. Wheelchair Accessible?	☐ Yes 1	☐ No 2	
168e. Additional Comments (e.g. equipment, etc.)?			

169. Other (specify primary function):			☐ N/A 97
169a. Secondary Function (if applicable)?			☐ N/A 97
169b. Size?	sq. m. or sq. ft.		
169c. Capacity (if visually available)?			☐ N/A 97
169d. Wheelchair Accessible?	☐ Yes 1	☐ No 2	
169e. Additional Comments (e.g. equipment, etc.)?			

170. Other (specify primary function):			☐ N/A 97
170a. Secondary Function (if applicable)?			☐ N/A 97
170b. Size?	sq. m. or sq. ft.		
170c. Capacity (if visually available)?			☐ N/A 97
170d. Wheelchair Accessible?	☐ Yes 1	☐ No 2	
170e. Additional Comments (e.g. equipment, etc.)?			

PART 9: OVERALL AUDITED BUILDING/HOUSING

Comfort/Accessibility

171. Is a map showing community resources available in a convenient public location?	□ Yes 1	□ No 2	
172. Is there a bulletin board in a public location?	□ Yes 1	□ No 2	
173. Is there a posted list of the staff?<br>173a. If so, does it include pictures?	□ Yes 1<br>□ Yes 1	□ No 2<br>□ No 2	 □ N/A 97
174. Is there a posted list of the residents?<br>174a. If so, does it include pictures?	□ Yes 1<br>□ Yes 1	□ No 2<br>□ No 2	 □ N/A 97
175. Is there a sound system or P.A. system?	□ Yes 1	□ No 2	
176. Is there a building security system?	□ Yes 1	□ No 2	□ N/A 97
177. Is there an air-conditioning system?	□ Yes 1	□ No 2	
178. Is there a gift shop, commissary, or store?	□ Yes 1	□ No 2	
179. Is there a kitchen area where a resident or visitor can make a cup of coffee, heat some soup, or the like?	□ Yes 1	□ No 2	
180. Are there vending machines for candy or soft drinks?<br>180a. If so, are they used by residents?	□ Yes 1<br>□ Yes 1	□ No 2<br>□ No 2	 □ N/A 97

181. Is there signage showing actions taken for COVID-19 safety protocols?<br>181a. Are the protocols posted on bulletin board?<br>181b. Do the protocols impact access to gift shop, commissary or store?<br>181c. Do the protocols impact access to kitchen?<br>181d. Do the protocols impact access to vending machine?<br>181e. Are the protocols posted in multiple languages?<br>181e.i. If yes, please note the languages below:	□ Yes 1<br>□ Yes 1<br>□ Yes 1<br>□ Yes 1<br>□ Yes 1<br>□ Yes 1	□ No 2<br>□ No 2<br>□ No 2<br>□ No 2<br>□ No 2<br>□ No 2	□ N/A 97<br>□ N/A 97<br>□ N/A 97<br>□ N/A 97<br>□ N/A 97<br>□ N/A 97<br>□ N/A 97

182. NOTES/COMMENTS:

SECTION C: RESIDENTS' UNITS
Control/Autonomy/Belonging/Comfort/Safety/Security/Identity

PART 1: RESIDENT UNIT – GENERAL LIVING SPACE

183. Are resident units/rooms single or shared occupancy?	☐ (Single 1)	☐ (Shared 2)	☐ N/A 97
183a. If shared, is there a room divider for individual privacy?	☐ Yes 1	☐ No 2	☐ N/A 97
184. Are the doors to the unit/room lockable?	☐ Yes 1	☐ No 2	
185. Are the doors to the unit/room equipped with a peephole?	☐ Yes 1	☐ No 2	☐ N/A 97
186. Does the unit/room have raised thresholds at the entrance?	☐ Yes 1	☐ No 2	
187. Does the front door have a lever or round handle?	☐ (Lever 1)	☐ (Round 2)	
188. Equipped with smoke detection devices?<br>188a. Fire extinguishers?<br>188b. Carbon Monoxide detectors?<br>188c. Sprinkler system?	☐ Yes 1<br>☐ Yes 1<br>☐ Yes 1<br>☐ Yes 1	☐ No 2<br>☐ No 2<br>☐ No 2<br>☐ No 2	☐ N/A 97<br>☐ N/A 97<br>☐ N/A 97<br>☐ N/A 97
189. Are the floors to the unit/room carpeted?	☐ Yes 1	☐ No 2	
190. Is there a lockable security safe available in the unit/room?	☐ Yes 1	☐ No 2	☐ N/A 97
191. Is there a functional thermostat in the unit/room?	☐ Yes 1	☐ No 2	☐ N/A 97
192. Are baseboard heaters present?<br>192a. Fireplaces?<br><br>(Note: If 'NO' for baseboard or fireplace heating, note the type of heating system used in Q238)	☐ Yes 1<br>☐ Yes 1	☐ No 2<br>☐ No 2	☐ N/A 97<br>☐ N/A 97
193. Is there a fan in the unit/room (e.g. ceiling or stationary)?	☐ Yes 1	☐ No 2	☐ N/A 97
194. Is the unit/room equipped with an area for socializing?<br>194a. Furniture that faces or is angled toward each other?	☐ Yes 1<br>☐ Yes 1	☐ No 2<br>☐ No 2	 ☐ N/A 97
195. Would you describe the furnishings/finishes as home-like?	☐ Yes 1	☐ No 2	☐ N/A 97
196. Does the unit have in suite laundry?	☐ Yes 1	☐ No 2	☐ N/A 97

PART 2: RESIDENT UNIT – KITCHEN SPACE

197. Is the unit/room equipped with an area for cooking?<br>197a. Stove-top?<br>197b. Oven?<br>197c. Fridge?<br>197d. Cupboard space?<br>197e. Microwave?	□ Yes 1<br>□ Yes 1<br>□ Yes 1<br>□ Yes 1<br>□ Yes 1<br>□ Yes 1	□ No 2<br>□ No 2<br>□ No 2<br>□ No 2<br>□ No 2<br>□ No 2	 □ N/A 97<br>□ N/A 97<br>□ N/A 97<br>□ N/A 97<br>□ N/A 97
198. Are the appliances accessible?	□ Yes 1	□ No 2	□ N/A 97
199. Is the kitchen counter height a minimum 71 cm from the floor?	□ Yes 1	□ No 2	□ N/A 97
199a. What is the height of the kitchen counter?	cm. or m.		
200. Is the unit/room equipped with an area for dining?	□ Yes 1	□ No 2	□ N/A 97
201. Is the unit/room lighting adequate?	□ Yes 1	□ No 2	
201a. How many light fixtures are present?	#		
201b. How many light fixtures are lit/functional?	#		□ N/A 97
202. Does this space have natural light?	□ Yes 1	□ No 2	
202a. If so, how many windows?	#		
202b. Do the windows open and close to allow ventilation?	□ Yes 1	□ No 2	□ N/A 97
203. Do the windows have a grill or guard rails on the outside?	□ Yes 1	□ No 2	□ N/A 97
204. Do the windows have blinds or drapes?	□ Yes 1	□ No 2	□ N/A 97

PART 3: RESIDENT UNIT – BATHROOM SPACE

205. Is the unit/room equipped with a private bathroom?	☐ Yes 1	☐ No 2	
206. Is the bathroom wheelchair accessible?	☐ Yes 1	☐ No 2	☐ N/A 97
207. Does the bathroom have raised thresholds at the entrance?	☐ Yes 1	☐ No 2	☐ N/A 97
208. Does the door have a lever or round handle?	☐ (Lever 1)	☐ (Round 2)	
209. Do the bathroom doors open out?	☐ Yes 1	☐ No 2	☐ N/A 97
210. Is there a showerhead?<br>210a. Is there a bathtub?	☐ Yes 1<br>☐ Yes 1	☐ No 2<br>☐ No 2	☐ N/A 97<br>☐ N/A 97
211. Are there handrails or safety bars in the bathtub?	☐ Yes 1	☐ No 2	☐ N/A 97
212. Are there lift bars next to the toilet?	☐ Yes 1	☐ No 2	☐ N/A 97
213. Are the towel racks a minimum height of 102 cm from the floor?	☐ Yes 1	☐ No 2	☐ N/A 97
213a. What is the height of the towel rack(s)?	cm. or m.		
214. Are the dispensers a minimum height of 38 cm from the floor?	☐ Yes 1	☐ No 2	☐ N/A 97
214a. What is the height of dispenser(s)?	cm. or m.		
215. Are there mirrors in the bathroom?	☐ Yes 1	☐ No 2	☐ N/A 97
216. Do areas subject to wetness have non-slip surfaces?	☐ Yes 1	☐ No 2	☐ N/A 97
217. Are there call buttons?	☐ Yes 1	☐ No 2	☐ N/A 97
218. Is there adequate lighting?	☐ Yes 1	☐ No 2	
218a. How many light fixtures are present?	#		
218b. How many light fixtures are lit/functional?	#		☐ N/A 97
219. Does this space have natural light?	☐ Yes 1	☐ No 2	
219a. If so, how many windows?	#		
219b. Do the windows open and close to allow ventilation?	☐ Yes 1	☐ No 2	☐ N/A 97
220. Do the windows have a grill or guard rails on the outside?	☐ Yes 1	☐ No 2	☐ N/A 97
221. Do the windows have blinds or drapes?	☐ Yes 1	☐ No 2	☐ N/A 97

PART 4: RESIDENT UNIT – BEDROOM SPACE

222. Is the unit/room equipped with a private bedroom for the resident (e.g. separate room):	☐ Yes 1	☐ No 2	
223. Does the entrance to the bedroom have raised thresholds at the entrance?	☐ Yes 1	☐ No 2	☐ N/A 97
224. Does the bedroom have a door?	☐ Yes 1	☐ No 2	☐ N/A 97
225. Does the front door have a lever or round handle?	☐ (Lever 1)	☐ (Round 2)	
226. Does the bedroom door lock?	☐ Yes 1	☐ No 2	☐ N/A 97
227. Do the bedroom door open out?	☐ Yes 1	☐ No 2	☐ N/A 97
228. Is the maximum height of the bed 46 cm?	☐ Yes 1	☐ No 2	☐ N/A 97
228a. Is the bed frame adjustable?	☐ Yes 1	☐ No 2	☐ N/A 97
229. Is the bedroom equipped with a closet space?	☐ Yes 1	☐ No 2	☐ N/A 97
230. Is there adequate lighting?	☐ Yes 1	☐ No 2	
230a. How many light fixtures are present?	#		
230b. How many light fixtures are lit/functional?	#		☐ N/A 97
231. Does this space have natural light?	☐ Yes 1	☐ No 2	
231a. If so, how many windows?	#		
231b. Do the windows open and close to allow ventilation?	☐ Yes 1	☐ No 2	☐ N/A 97
232. Do the windows have a grill or guard rails on the outside?	☐ Yes 1	☐ No 2	☐ N/A 97
233. Do the windows have blinds or drapes?	☐ Yes 1	☐ No 2	☐ N/A 97
234. What is the size of the bedroom?	sq. m. or sq. ft.		☐ N/A 97

235. Variation in design of residents' units:	☐ N/A 97
As if effort was made to vary style and décor from room to room	☐ Distinct Variation 1
Rooms/apartments are distinct, but there is a general décor throughout	☐ Moderate Variation 2
Some variation in size, shape, and furniture arrangement; not noticeable unless looked for.	☐ Nearly Identical 3

PART 5: PERSONALIZATION

236. Personalization within residents' units:<br>(please choose one of the 4 options below)	☐ N/A 97
Most of the furnishings and objects in the rooms belong to the individual; time and energy have been spent in personalizing the rooms	☐ Much Personalization 1
Residents have added personal objects such as rugs, pictures, chairs, favorite objects	☐ Some Personalization 2
Some family pictures or personal articles, but room does not seem to belong to individual	☐ Little Personalization 3
No personalization is evident	☐ No Personalization 4

237. Personalization of entryways residents' units:<br>(please choose one of the 4 options below)	☐ N/A 97
Distinctly different treatment of each entryway; decorated with different personal objects	☐ Much Personalization 1
e.g. Doorways painted different colours; individualized nameplates to identify resident	☐ Some Personalization 2
e.g. Some variation in colour schemes, or nameplates only	☐ Little Personalization 3
No personalization is evident	☐ No Personalization 4

238. NOTES/COMMENTS:

SECTION D: CONCLUDING RATING: PROJECT/HOUSING COMPLEX DÉCOR and APPEARANCE

239. Overall distinctiveness of the housing complex:<br>(please choose one of the 3 options below)	
A concerted effort has been made to vary the décor from room to room	☐ Much Distinctiveness 1
Furnishings vary from room to room, but the overall room design is the same; wall texture and floor coverings show little variation	☐ Moderate Distinctiveness 2
Institutional appearances; most areas are quite similar, as in hospital (without furniture, all rooms look similar)	☐ Little Distinctiveness 3
