# Supplementary material for: Aging in the Right Place for Older Adults Experiencing Housing Insecurity: An Environmental Assessment of Temporary Housing Program
Source: Int J Environ Res Public Health. 2022 Nov 11;19(22):14857. doi: 10.3390/ijerph192214857 (PMC9691151; doi:10.3390/ijerph192214857)
Supplement: Supplementary file 1 [file ijerph-19-14857-s001.zip › 2021.03.11_AIRP-ENV-SO Tool.pdf]

AGING IN THE RIGHT PLACE
SECONDARY OBSERVATION OF THE ENVIRONEMENT (AIRP-ENV Pt.2)

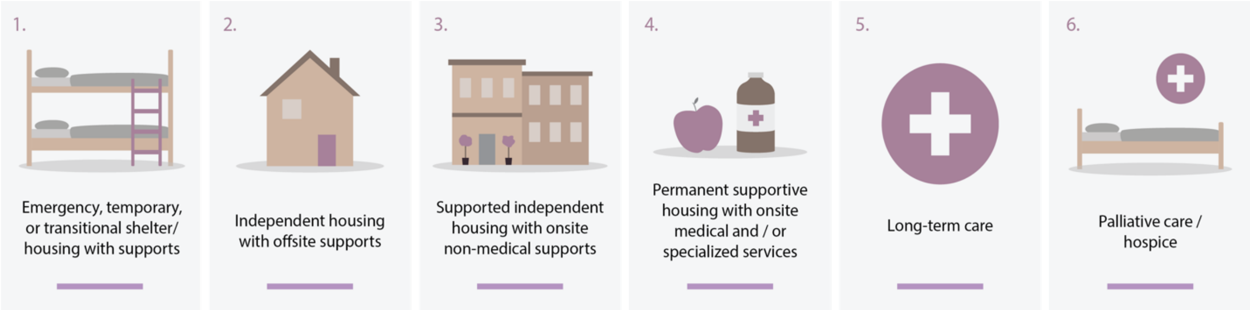

PROJECT TITLE:
Aging in the Right Place: Building Capacity for Promising Practices that Support Older People Experiencing Homelessness in Montreal, Calgary, and Vancouver

Project Director:
Dr Sarah Canham, College of Social Work, University of Utah; Gerontology Department., Simon Fraser University, scanham@sfu.ca
Vancouver City-Lead
Dr. Atiya Mahmood, Associate Professor, Gerontology Department, Simon Fraser University, amahmood@sfu.ca
Calgary City-Lead
Dr. Christine Walsh, Professor, Faculty of Social Work, University of Calgary, cwalsh@ucalgary.ca
Montreal City-Lead
Dr. Tamara Sussman, Associate Professor, School of Social Work, McGill University, tamara.sussman@mcgill.ca

GRANTING AGENCY:
Canadian Mortgage and Housing Corporation - Social Sciences and Humanities Research Council

AGING IN THE RIGHT PLACE ENVIRONMENTAL AUDIT TOOL (AIRP- ENV PT 2.)

Introduction

Secondary observations to collect information regarding the immediate surrounding environment of the promising practice organizations. The RA will spend 15 to 25 minutes looking at 1-3 blocks around the promising practice organization to collect the following information. The collected information can be complimented by google searches on features (i.e. how busy are public spaces) to get a holistic snapshot.

Date of Data Collection: ______________________________

Start Time (AM/PM): ____________________ End Time (AM/PM): ____________________

Break (minutes – if applicable): ______________________________

Name of Research Assistant: ______________________________

Name of Promising Practice: ______________________________

# of Building Location(s) used for Promising Practice: ______________________________

Identifying Name of Audited Building: ______________________________

Address of Audited Building: ______________________________

Q1: What are the predominant land uses (i.e., residential, commercial, industrial, or mixed)?

Q2: What are the characteristics of this area?

[Make a note of the maintenance or upkeep of the buildings, street & sidewalks, availability of shelter or unsheltered transit stops, presence of features like curb cuts, stops signs, traffic lights, gentrification/ change in land use, and support services (such as pharmacies, safe injection sites etc.)]

Q3: Describe the quality of public spaces/amenities in the area. How do they support social interaction and public.

[Observe the type of public spaces located in the area (restaurants, parks, library, banks, religious/spiritual places, etc.) and whether they are accessible, types of uses/activities and users (i.e., age groups) these public spaces supports, the extent to which these public spaces are being used, and whether public spaces are even present in the area?]

Q4: How safe walking in this area feels like? Consider both physical and social indicators (i.e. presence and quality of street life).

[Make note of 1) physical aspects like graffiti/vandalism/garbage/ hazardous material, litter, windows with bars, signs of substance abuse, abandoned buildings, absence of streetlights, and 2) Social aspects like frequent conflict between users of the space].

Q5: How convenient is it for the users of this area to travel through it? Are they able to get where they want to get to?

[Consider street connectivity, presence of sidewalk, traffic safety that supports walking/wheeling on the sidewalk and street crossings. Specify areas with noticeable issues, if applicable].

Q6: Describe the overall traffic volume, speed and noise in this area?

[Make note of does this area's predominant traffic volume (heavy, moderate, or light), how quickly do cars travel in this area, level of traffic noise, and other noises (e.g., industrial, birds, dogs, loud music), if applicable].

Q7: Additional comments:
